# Long-term effects of the terror attack in Berlin in 2016 on paranoid ideation in female emergency personnel

**DOI:** 10.1192/bjo.2020.57

**Published:** 2020-08-03

**Authors:** Ulrich Wesemann, Manuel Mahnke, Sarah Polk, Gerd Willmund

**Affiliations:** Psychotrauma Center, German Armed Forces Hospital Berlin, Germany; Psychotrauma Center, German Armed Forces Hospital Berlin, Germany; and Fire and Rescue Station Wedding, Voluntary Fire Brigade, Germany; Max Planck Institute for Human Development, Germany; Psychotrauma Center, German Armed Forces Hospital Berlin, Germany

**Keywords:** Terror attack, paranoid ideation, emergency personnel, gender, trauma

## Abstract

In a pilot study, female emergency personnel showed increased paranoid ideation following a terror attack. This newly designed confirmatory study aims to replicate these previously found gender-specific results and investigate the progression of effects after 2 years. Participants were exposed and unexposed emergency personnel (*n* = 120). Exposed female versus exposed male personnel showed higher paranoid ideation at both time points. There was a group × time interaction effect in paranoid ideation: paranoid ideation increased over time in the exposed versus the unexposed female group. The same effect was observed with exposed female emergency personnel showing a significant 2-year post-deployment increase compared with the total group including unexposed female as well as exposed and unexposed male emergency personnel. There is, as yet, no conclusive explanation for this difference. Sexual harassment in a male-dominated profession may be a vulnerability factor. Differentiated preparation and follow-up for emergency responders is recommended moving towards health-related equality.

## Background

Responses to a potentially traumatic event span a wide range from increased stress to mental disorders, such as depression, post-traumatic stress disorder, other anxiety disorders or addiction.^1^ Numerous publications focus on the psychological effects of disasters on emergency responders, but the systematic investigation of differences between genders or occupational groups is comparatively limited.^2^ Indeed, the psychological reactions of emergency personnel exposed to terror attacks in Europe have until now rarely been examined.^3–5^ However, understanding these differences is necessary for the development of specific preparation and follow-up tools. In particular, gender differences are of interest in addressing health inequalities in pre- and post-mission outcomes.

In military contexts, computer-based prevention programs that are target group-specific can be used during preparation for foreign missions; in the USA, the ‘Stress Resilience in Virtual Environments Program’ (STRIVE) was developed for this purpose.^6^ STRIVE is specific to target group and deployment, although not adaptive because of its fixed programmed scenarios. In Germany, the ‘Chaos Driven Situation Management Retrieval System’ (CHARLY) was recently launched. CHARLY is a blended learning program that has already been made available for certain military professions, such as paramedics or ordnance technicians, and has demonstrated a specific preventive effect on post-traumatic stress disorder.^7^ Similarly, specific programs would be useful for emergency personnel.

## Research findings after the 2016 terrorist attack in Berlin

In order to examine whether the efforts to investigate occupation- and gender-specific differentiation is warranted, an explorative pilot study was conducted after the 2016 terrorist attack in Berlin, when a tractor-trailer was steered into a crowd at the Breitscheidplatz Christmas market. This was the first Islamist terrorist attack in Germany in which more people died than the perpetrator himself. In total, 12 people were killed and another 55 injured. Initial cross-sectional analyses found occupational and gender differences; notably, female personnel showed higher paranoid ideation.^8^ Although the results regarding the occupational groups have been replicated (U. Wesemann, personal communication, 2020), those regarding gender-specific differences in paranoid ideation are still provisional and no conclusive explanation for this difference is given. A meta-analysis showed a robust association between paranoid ideation and violence in the general population, and this association remained stable when controlling for drug misuse or other psychiatric comorbidities.^9^ Regarding gender differences, the theory of tokenism predicts gender stereotypes and harassment in organisations where women are underrepresented.^10^ This theory seems to be of particular relevance in organisations where length of employment is shorter for women,^11^ which is often the case in emergency services such as fire brigades. Indeed, sexual assaults on women are more common in male-dominated occupations,^12^ which could increase vulnerability to paranoid ideation. Therefore, this confirmation study aims to repeat these gender-specific findings, with the hypothesis of more pronounced paranoid ideation among exposed female emergency personnel.

## Method

All emergency personnel from the fire brigade, police, non-governmental organisations and civil rescue services deployed to respond to the terror attack at Breitscheidplatz in Berlin were given the opportunity to participate in the study (exposed group). A comparison group comprised personnel from the same units who had not responded to the attack. The recruitment was conducted with the consent of the organisations. Task force leaders were informed about the study and invited to participate. If task force leaders agreed, brief information sessions were held for the respective units, and attendees were informed about the purpose of the study. Participation in the information sessions and the study was voluntary, and written informed consent was given by all participants. The study was approved by the Ethics Committee of the Charité. The first survey was distributed 3–4 months after the attack, and a second measurement was conducted 18 to 21 months after the first survey. Participants received the questionnaires in the mail.

A total of *n* = 120 volunteers participated at the first time point, 60 in the exposed group and 60 in the comparison group. At the second time point, 39 individuals from the exposed group and 33 from the comparison group responded. All deployed personnel had been on site with the exception of a dispatcher. Gender and occupational group distributions are shown in Table 1.

**Table 1:** Distribution of emergency service personnel according to gender, occupation and deployment to the terror attack

Deployment at Breitscheidplatz	Fire Brigade	Armed Forces	NGO	Police	PSNV	Total
Yes						
Male	22	2	9	5	4	42
Female	2	0	4	5	6	17
Other						1^a^
Total	24	2	13	10	10	60
No						
Male	21	0	0	17	0	38
Female	0	0	0	20	2	22
Total	21	0	0	37	2	60
Total						
Male	43	2	9	22	4	80
Female	2	0	4	25	8	39
Other						1^a^
Total	45	2	13	47	12	120

NGO, non-governmental organisation; PSNV, Psychosocial Emergency and Crisis Network (Psychosoziale Notfallversorgung);

a. Excluded from occupational affiliation because of data protection reasons.

Data collection consisted of questionnaires including demographics and the Brief Symptom Inventory (BSI) to evaluate paranoid ideation. The BSI consists of five items that assess aspects of mistrust up to strong paranoid ideation on a five-point Likert scale.^13^

Statistical analyses were conducted with SPSS (Version 21). Missing values at the second time point were imputed using the baseline-observation-carried-forward method. To test for drop-out bias, two-sample *t*-tests were conducted for metric parameters and Pearson χ^2^ was calculated for categorial parameters. Using linear regression, the exposed group and comparison group were tested separately to test whether gender had an influence on paranoid ideation. Participants who indicated their gender as ‘other’ were excluded, as the small number (*n* = 1) would not have allowed for meaningful evaluation. In order to check whether the occupational group had an influence on paranoid ideation, a Kruskal–Wallis *H*-test was carried out within deployed female personnel. Additionally, differences in change in paranoid ideation between the exposed group and comparison group were examined using a single-factor repeated measures ANOVA (rmANOVA) with a Bonferroni *post hoc* test.

## Results

There were no significant differences between the completers and those who dropped out (Table 2). Linear regression analysis revealed a significant influence of gender on paranoid ideation within the exposed group, *F*(1,57) = 11.6, *P* = 0.001, *R*^2^ = 0.17, β = 0.41, with female personnel scoring higher. This influence was not seen within the comparison group, *F*(1,58) = 3.1 (not significant). Similar results were found 2 years after the attack: female personnel in the exposed group continued to report higher paranoid ideation, *F*(1,57) = 8.9, *P* = 0.004, *R*^2^ = 0.14, β = 0.37. This influence was absent in the comparison group, *F*(1,57) = 1.6 (not significant). rmANOVA revealed an interaction effect in paranoid ideation, *F*(1, 115) = 5.0, *P* = 0.027, ƞ² = 0.04; compared with all other participants, paranoid ideation increased significantly among exposed female personnel. This interaction effect remained when directly comparing only female personnel of each group, *F*(1, 37) = 4.2, *P* = 0.047, ƞ² = 0.103, with a significant increase in paranoid ideation in exposed female personnel. Finally, a Kruskal–Wallis *H*-test carried out within deployed female personnel showed that there was no statistically significant difference in paranoid ideation score between the different occupation groups, χ²(3) = 1.92, *P* = 0.589.

**Table 2:** *t*-test showing no significant differences between the participants who responded at both time points and those who responded at baseline only

	Both time points			Baseline only					
	*n*	Mean	s.d.	*n*	Mean	s.d.	*t*	d.f.	*P*
Paranoid ideation	72	1.38	1.38	48	1.15	1.34	0.90	118	0.368
Rank	39	1.64	0.71	28	1.39	0.50	1.69	65	0.116
Years of service	69	14.74	12.04	46	14.30	10.47	0.20	113	0.842
Age, years	69	40.12	11.19	46	39.57	11.09	0.26	113	0.796

Cisgender: χ²_(1, *n* = 119)_ <1; not significant.

## Discussion

The current study replicated the results of the pilot study. The finding that gender influences paranoid ideation following terror attacks or catastrophes has, to our knowledge, not been replicated in other studies since, but studies relating high-risk occupations to paranoid ideation are so far inconsistent. For example, medical prison staff reported higher paranoid ideation than the general population,^14^ but veterans deployed to the Gulf War did not differ from a matched comparison group of soldiers.^15^ However, these studies did not control for gender, and although in the sample of medical prison staff, the proportion of female employees (53.6%) may have driven this difference, among the sample of Gulf War veterans, only 3.1% were women. Thus, future research may consider gender-specific evaluations, or controlling for gender when comparing different studies.

As a result of limitations of this study, the results should be interpreted carefully. The sample size for the subgroup analysis was small, and because of a drop-out rate of 40%, some data had to be imputed at the second time point, which notably restricts interpretation of the effects seen after 2 years. Further, because of the small sample size, direct comparison between female personnel in the exposed group and comparison group was only carried out over time. Also, approximately 90% of the female control group are police, limiting representativeness. Nevertheless, occupational group differences for paranoid ideation were ruled out for deployed female emergency responders.

Despite these limitations, the replication of the results of the pilot study provides an indication of gender-specific effects. The current study suggests that there is an elevated need for improvement in health-related gender equality. Our results could be used for the development of a gender-specific module before and after the deployment.

## Data Availability

The data that support the findings of this study are available from the corresponding author upon reasonable request.
